# Application of optical coherence tomography enhances reproducibility of arthroscopic evaluation of equine joints

**DOI:** 10.1186/1751-0147-56-3

**Published:** 2014-01-10

**Authors:** Tytti Niemelä, Tuomas Virén, Jukka Liukkonen, David Argüelles, Nikae C R te Moller, Pia H Puhakka, Jukka S Jurvelin, Riitta-Mari Tulamo, Juha Töyräs

**Affiliations:** 1 Department of Equine and Small Animal Medicine, University of Helsinki, Helsinki, Finland; 2 Cancer Center, Kuopio University Hospital, Kuopio, Finland; 3 Department of Applied Physics, University of Eastern Finland, Kuopio, Finland; 4 Department of Equine Sciences, Faculty of Veterinary Medicine, Utrecht University, Utrecht, the Netherlands; 5 Department of Clinical Neurophysiology, Kuopio University Hospital, Kuopio, Finland

**Keywords:** Arthroscopy, Cartilage lesion, Horse, ICRS-scoring, Optical coherence tomography

## Abstract

**Background:**

Arthroscopy is widely used in various equine joints for diagnostic and surgical purposes. However, accuracy of defining the extent of cartilage lesions and reproducibility in grading of lesions are not optimal. Therefore, there is a need for new, more quantitative arthroscopic methods. Arthroscopic optical coherence tomography (OCT) imaging is a promising tool introduced for quantitative detection of cartilage degeneration and scoring of the severity of chondral lesions. The aim of this study was to evaluate the inter-investigator agreement and inter-method agreement in grading cartilage lesions by means of conventional arthroscopy and with OCT technique. For this aim, 41 cartilage lesions based on findings in conventional and OCT arthroscopy in 14 equine joints were imaged, blind coded and independently ICRS (International Cartilage Repair Society) scored by three surgeons and one PhD-student.

**Results:**

The intra- and inter-investigator percentages of agreement by means of OCT (68.9% and 43.9%, respectively) were higher than those based on conventional arthroscopic imaging (56.7% and 31.7%, respectively). The intra-investigator Kappa coefficients were 0.709 and 0.565 for OCT and arthroscopy, respectively. Inter-investigator Kappa coefficients were 0.538 and 0.408 for OCT and arthroscopy, respectively.

**Conclusions:**

OCT can enhance reproducibility of arthroscopic evaluation of equine joints.

## Background

Prevalence of severe cartilage lesions in equine joints is high, especially in racehorses. The lesions can lead to post-traumatic osteoarthritis and have major effect on the career of the horse [1]. Accurate grading of chondral lesions is needed in order to choose the optimal treatment and to set reliable prognosis. In addition to the ICRS (International Cartilage Repair Society) scoring system [2], lesion area and correction factor for weight bearing have been used in evaluation of the severity of equine articular cartilage injuries in experimental settings [1]. In equine arthroscopies, modified inflammatory and degenerative scores have also been used [3].

In conventional arthroscopic evaluation degenerative changes present below a visually intact cartilage surface cannot be detected. Furthermore, differentiation of superficial and deep cartilage lesions is challenging without accurate knowledge on their relative depth [4]. In a recent survey roughly half of the highly experienced arthroscopic surgeons considered that assessment of severity of cartilage lesions needs improvement [5]. Furthermore, in a prospective blinded study conducted by four experienced arthroscopic surgeons, poor inter- and intraoperator reproducibility of conventional arthroscopic evaluation of cartilage injuries in human knee was reported [4].

In some equine joints (e.g. distal interphalangeal joint) significant part of the articular surface cannot be reached and assessed with conventional arthroscopy. An imaging technique that could provide improvement for arthroscopic assessment and accessibility of articular cartilage areas is optical coherence tomography. Optical coherence tomography (OCT) is a diagnostic imaging technique commonly used in human cardiovascular surgery and ophthalmology. OCT is based on the measurement of reflection and backscattering of near infrared light from tissues [6]. It provides cross-sectional images of three dimensional objects at resolutions comparable to that of low-power microscopy. Small diameter of OCT catheter enables minimal invasive diagnostic procedures. It has been shown to be feasible for evaluation of bovine articular cartilage *in vitro*[7], as well as human cartilage during arthroscopy *in vivo*[8] and *in vitro*, where findings in OCT examinations were shown to correlate well with structural details seen under histological evaluation [9-11].

In a study by Chu et al. [8] arthroscopic optical coherence tomography (OCT) imaging of human knee joint frequently revealed surface and subsurface abnormalities which were not detected by means of conventional arthroscopy. This was also seen in a recent equine study where OCT arthroscopies were conducted by insertion of a thin flexible catheter in the metacarpophalangeal joint [12]. In addition, arthroscopically inaccessible areas could be reached by the OCT catheter in some cases. Therefore, arthroscopic OCT could increase the sensitivity of diagnostics over a larger surface area compared with conventional arthroscopy.

Optical coherence tomography may have potential for arthroscopic evaluation of cartilage lesions in various equine joints. However, it has not been previously applied to evaluate cartilage in equine distal interphalangeal, intercarpal, tarsocrural or stifle joints. We hypothesize that ICRS scoring of cartilage lesions is more reproducible when based on OCT imaging than when based on conventional arthroscopy. In the present study this hypothesis is tested by conducting conventional and OCT arthroscopies in a total of 14 equine joints to determine the intra- and inter-investigator and inter-method agreement in ICRS scoring of 41 arthroscopy and 41 OCT images retrospectively.

## Methods

Three surgeons and one PhD-student scored the cartilage lesions in blind-coded arthroscopic (n = 41) and corresponding OCT images (n = 41) in random order. Each investigator repeated scoring of the 82 images for three times. Arthroscopic and OCT images were captured from videos recorded during arthroscopy of cadaver joints. These joints of fore- and hindlimbs of donated euthanized adult horses, previously used for racing purposes, were obtained from Veterinary Teaching Hospital of University of Helsinki and frozen at -20°C until conventional and OCT arthroscopies were performed.

Standard diagnostic arthroscopies of the most commonly evaluated joints and approaches in clinical settings were conducted using a 4 mm, 30° arthroscope (Karl Storz GmbH & Co, Tuttlingen, Germany) [13]. These were dorsal aspects of the distal interphalangeal joint, dorsal and palmar/plantar aspects of the metacarpo-/metatarsophalangeal joint, dorsal aspects of the intercarpal and tarsocrural joint and cranial aspect of the medial femorotibial joint and femoropatellar joint (Table 1).

**Table 1:** Number of scored lesions (N) in variable sites of different joints (Mc/Mt = Metacarpal/Metatarsal bone)

**Joint**	**Site in the joint**	**N**
Intercarpal	Third carpal bone	3
Intercarpal	Os carpi radialis	4
Metacarpo-/Metatarsophalangeal	Dorsal sagittal ridge of the Mc/Mt3	3
Metacarpo-/Metatarsophalangeal	Dorsal medial/lateral condyle of the Mc/Mt 3	6
Metacarpo-/Metatarsophalangeal	Dorsal proximal eminence of the proximal phalanx	1
Metacarpo-/Metatarsophalangeal	Palmar/plantar sagittal ridge of the Mc/Mt3	4
Metacarpo-/Metatarsophalangeal	Dorsal aspect of medial/lateral sesamoid bone	10
Tarsocrural	Trochlear ridge of the talus	2
Tarsocrural	Distal intermediate ridge of the tibia	1
Femoropatellar	Trochlear ridge of the femur	3
Femoropatellar	Trochlear groove of the femur	3
Femoropatellar	Patella	1

**Figure 1:**
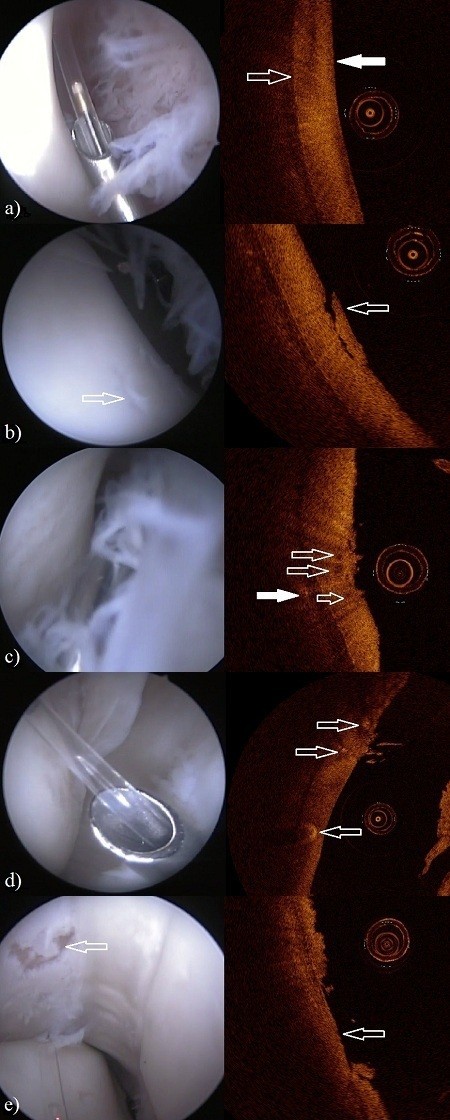
**The arthroscopy (left) and OCT (right) images of the metacarpo-/metatarsophalangeal joint.** The circular object in OCT images is the OCT catheter (diameter = 0.9 mm). **a)** Dorsal aspect of the lateral sesamoid bone. In the arthroscopic view the tip of the custom made hollow instrument with OCT catheter lie on the examined cartilage surface (ICRS score 0). In the OCT image the articular surface (white arrow) and the interface between cartilage and subchondral bone are visible (open arrow). **b)** In the arthroscopic view cartilage of the dorsal aspect of the lateral sesamoid bone with lesion (open arrow) is visualized, synovial villi can be seen in the right. The OCT image shows the same lesion (ICRS score 2) with cartilage flap (open arrow) still attached. **c)** In the arthroscopic view cartilage of the dorsal aspect of the medial sesamoid bone with roughening of the surface is in the left, synovial villi are in the right. The OCT image shows the fibrillation with high definition (ICRS score 2). Beneath the cartilage surface there is cavitation (open arrows) and within the subchondral bone an anomaly (white arrow) can be seen. **d)** In the view of an arthroscope dorsolateral condyle of third metacarpal bone with cartilage lesion below the OCT catheter can be seen. In the OCT image the lesion (ICRS score 1) and also anomalies beneath the cartilage surface are visible (arrows). **e)** Severe lesion (ICRS score 4, arrow) of medial sesamoid bone with complete loss of cartilage can be seen in both arthroscope and OCT images.

Identified cartilage lesions were evaluated by conventional arthroscopic examination and OCT imaging. After visualization of lesion with the arthroscope, the OCT catheter (diameter = 0.9 mm) was guided into the joint through a custom made hollow instrument under arthroscopic control (Figure 1). OCT catheter was set over the visually deepest spot of each lesion. OCT imaging was conducted using the ILUMIEN PC1 Optimization System (St. Jude Medical; St. Paul, Minnesota, USA: wavelength 1305 ± 55 nm, axial resolution ≤ 20 μm, lateral resolution 25–60 μm, slice thickness 100 μm, frame rate 100 frames/s). Representative arthroscopy videos and OCT recordings were selected and still images were captured for retrospective analysis.

In the distal interphalangeal joint the area of cartilage surface under evaluation was increased by applying the OCT catheter via dorsal portal between the surfaces of middle and distal phalanx until the navicular bone was reached. Images captured this way were not included in the retrospective scoring of injuries since arthroscopic views were not available.

The images were captured so that arthroscopic view would best show the entire lesion of interest and the OCT images would show the most severe spot of this particular lesion. To ensure that scoring was conducted at the same spot with both techniques the lesion of interest was indicated by an arrow or circle in the images. International Cartilage Repair Society (ICRS) scores were used for grading, classifying cartilage lesions in four grades; grade 0 (normal), grade 1 (superficial softening and/or superficial fissures and cracks), grade 2 lesions (lesions extending down to < 50% of cartilage depth), grade 3 lesion (severely abnormal with lesions extending > 50% of cartilage depth) and grade 4 (complete loss of cartilage, lesions extending through the subchondral bone plate [2]).

The percentages for agreement of the ICRS scores were determined to assess the agreement within and between investigators and imaging modalities. The intra-investigator and inter-investigator agreement was further evaluated with generalized Kappa coefficients. The intra-investigator Kappa coefficients were calculated separately for each investigator as well as an average over the four investigators. Furthermore, differences between the imaging methods in the grading of cartilage lesions were investigated with the mixed cumulative logit model. Differences between the methods were quantified with odds ratios (OR) and their 95% confidence intervals (CI). P < 0.05 was set as the limit for statistical significance. SAS® System for Windows, version 9.3 (SAS Institute Inc., Cary, NC, USA) and R for Windows, version 2.11.1 (R Foundation for Statistical Computing, Vienna, Austria) were used for statistical analysis.

## Results

**Figure 2:**
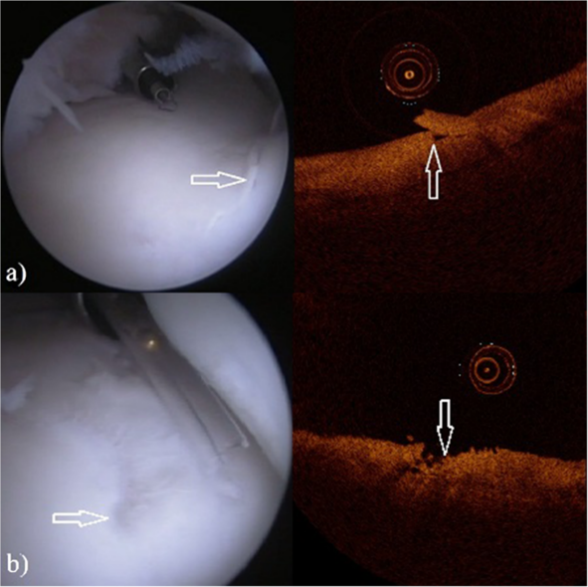
**The arthroscopy (left) and OCT (right) images of the third carpal bone in the intercarpal joint. a)** Lesion (arrows) in the arthroscopic image (left) and in the OCT image (right) with ICRS score of 2. **b)** Lesion (arrows) in the arthroscopic image (left) and in the OCT image (right) with ICRS score of 3.

Optical coherence tomography enabled detection of cartilage lesions of all ICRS scores. Furthermore, anomalies beneath the cartilage surface and within subchondral bone could also be visualized (Figures 1 and 2).

The average intra-investigator percentages of agreement were 68.9% and 56.7% for OCT and arthroscopy, respectively (Table 2). The intra-investigator Kappa coefficients were 0.709 and 0.565 for OCT and arthroscopy, respectively (Table 3).

**Table 2:** Individual and average (in bold) intra-investigator percentages of agreement. Each investigator scored independently 41 blind coded lesions in 82 images for three times

		**Agreeing scores**
**Imaging method**	**Investigator**	**%**
OCT	1	78.0
	2	63.4
	3	63.4
	4	70.7
	Average	**68.9**
Arthroscopy	1	58.5
	2	65.9
	3	48.8
	4	53.7
	Average	**56.7**

**Table 3:** Generalized Kappa (κ) coefficients for reproducibility of ICRS-score

**Imaging method**	**Scorings/method**	**Scorings/investigator**	**Lesions**	**Investigators**	**κ within investigators**	**κ between investigators**
OCT	164	3	41	4	0.709	0.538
Arthroscopy	164	3	41	4	0.565	0.408

The inter-investigator percentage of agreement was 43.9% for OCT scorings and 31.7% for arthroscopy scorings (Table 4). The inter-investigator Kappa coefficients were 0.538 and 0.408 for OCT and arthroscopy, respectively (Table 3). With OCT the probability of getting higher cartilage damage scores was higher than that of conventional arthroscopy, although difference was not statistically significant (odds ratio = 1.96, 95% confidence interval 0.83-4.63, p = 0.123, Table 5).

**Table 4:** Inter-investigator agreement of conventional arthroscopic and OCT evaluation

	**Agreeing scores**
**Imaging method**	**%**
OCT	43.9
Arthroscopy	31.7
Total	37.8

**Table 5:** Distribution of 82 median ICRS scores based on OCT or arthroscopy images (N = number of scored lesions)

	**0**		**1**		**2**		**3**		**4**	
**Imaging method**	**N**	**%**	**N**	**%**	**N**	**%**	**N**	**%**	**N**	**%**
OCT	12	29.3	12	29.3	11	26.8	3	7.3	3	7.3
Arthroscopy	12	29.3	18	43.9	8	19.5	2	4.9	1	2.4
Total	24	29.2	30	36.6	19	23.2	5	6.1	4	4.9

## Discussion

In this study reproducibility of ICRS scoring of cartilage lesions based on OCT and conventional arthroscopic images was investigated. In addition, the feasibility of OCT imaging at anatomic sites not achievable with conventional arthroscopy was evaluated.

**Figure 3:**
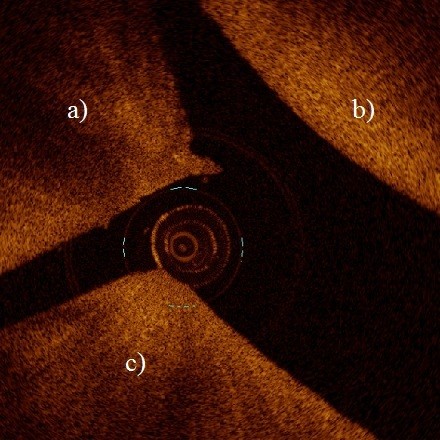
**OCT enables access to the palmar and plantar areas in distal interphalangeal joint which is not achievable with conventional arthroscopy.** Image shows the palmar aspect of the distal interphalangeal joint where dorsal aspect of the navicular bone **(a)** and part of the second **(b)** and third **(c)** phalanx are visible.

In general, the intra-investigator reproducibility was moderate to good and inter-investigator reproducibility only fair to moderate [14]. However, OCT was found to enable more reproducible scoring of lesions than conventional arthroscopic imaging. In addition, the best inter-investigator agreement was also based on the OCT images which supports our hypothesis about superior reproducibility of OCT based scoring when compared with conventional arthroscopic evaluation. Furthermore, articular surfaces in palmar or plantar pouches of distal interphalangeal joints were found to be accessible for OCT imaging (Figure 3).

In addition to more reproducible ICRS scoring, cross-sectional OCT images allow detection and classification of subsurface lesions [12]. Furthermore, OCT images have been shown to correspond well to histology of cartilage. In addition to ability to detect progressive articular cartilage thinning and disruption of the cartilage/bone interface confirmed in histology [11], OCT has been shown to correlate almost perfectly to histomorphometry [9]. Moreover, OCT enables detection of the earliest changes of osteoarthritis, such as hypocellularity of cartilage and changes in collagen organization, based on changed polarization properties of cartilage [10].

In the present study, scorings based on OCT imaging were found to be higher on average than scorings based on conventional arthroscopy, although this was not statistically significant finding. In the study of Kaleva et al. [15] higher ICRS scores were also received when arthroscopic ultrasound imaging was applied in human knee joints *in vivo*. Arthroscopic ultrasound imaging, as well as OCT, is able to detect early osteoarthritic changes, such as initial fibrillation of the articular surface [16-19]. Furthermore, as the thickness of cartilage is visible in cross sectional images, the relative depth of lesion may be evaluated by means of OCT or ultrasound imaging, which can considerably affect scoring of lesion severity. As arthroscopic OCT can be conducted through normal portals made for arthroscopy, it is not more invasive than conventional arthroscopy.

**Figure 4:**
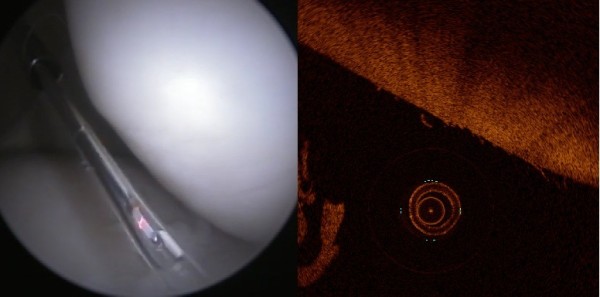
Mild cartilage fibrillation on the surface of the patella (ICRS score 1) visible only in OCT image (right).

All four investigators agreed in only 31-44% of arthroscopy and OCT images (depending on the method), which was overall a bit lower than the average intra-investigator agreement. However, the inter-investigator agreement reported here is higher than 17.4% agreement reported for conventional arthroscopic evaluation by Spahn et al. [4]. This inter-investigator discrepancy in scoring of lesions based on conventional arthroscopic images seen in this study as well as the results reported by Spahn et al. [4] emphazises the value of quantitative OCT imaging during arthroscopy. However, even with superior sensitivity of OCT to detect lesions, differentiation especially between grade 0 and 1 lesions and between grade 1 and 2 lesions was challenging. Some of the cartilage lesions were visible only in OCT images (Figure 4).

It is highly likely that agreement between investigators would have been higher provided that the real time OCT imaging had been available, and when combined with conventional arthroscopy to result even more reproducible scorings. Accurate evaluation of lesion depth would be important as the grade of the lesion affects not only the surgeon’s decision about treatment but also the prognosis, and thus sometimes even the survival of the horse. Based on the present results and earlier reports, combining the conventional arthroscopic examination with OCT could result in more accurate assessment [7,10,20,21]. Conventional arthroscopy could be used as a tool for general evaluation of the joint and guiding of multimodal imaging instruments which provide information of lesion depth and integrity of subsurface structures. When lesions are detected early, they can be treated in time when possible or decision of retirement of the horse to lighter work can be done to prevent further damages. Accuracy of diagnosis and better agreement between veterinarians is also needed to enhance objectivity and reliability of veterinary surgeon profession since the second opinion about diagnosis and prognosis is commonly requested by the clients.

Many areas in various equine joints are not achievable with conventional arthroscopy. For example, the dorsal pouch of the distal interphalangeal joint represents about 30% of the entire joint area and arthroscopy of the palmar/plantar pouch of the joint is even more limited [13], so that visualization of dorsal part of the navicular bone with the arthroscope is impossible. In the metacarpo-/metatarsophalangeal joint, the articular surface of the proximal phalanx, except from the dorsal and palmar margins, is not achievable. Still those areas in joints of racehorses often contain significant lesions which are not detected with conventional arthroscopy. Although they may not be surgically treatable either, neglecting them jeopardizes the accuracy of the prognosis. Although not set as the primary aim of this study, accessibility of distal interphalangeal joint with OCT catheter was tested. We managed to apply OCT catheter far on the plantar/palmar articular surfaces of distal interphalangeal joints until the dorsal part of the navicular bone was reached (Figure 3). Accessibility of narrow joint spaces is an important aspect of OCT arthroscopy and warrants further investigation.

This study had some limitations. The study design was retrospective and grading was based on blind coded still images of cartilage lesions. Therefore, it was not as optimal as in the true *in vivo* situation where the surgeon may evaluate and examine the lesions in a variety of views and distances. However, this limitation affects both evaluated techniques, thus minimizing its effect on the conclusions of the present study. In the present study, only reproducibility of ICRS scoring with conventional arthroscopy and OCT was evaluated. Thus, accuracy of scoring still remains unknown and it should be investigated in the future. Furthermore, development of computerized image recognition based scoring of quantitative OCT images could aid surgeon to achieve more objective and reproducible scoring.

OCT arthroscopy was conducted in previously frozen and thawed cadaver joints. This could have caused microdamage on cartilage tissue potentially affecting light reflection and scattering. However, usage of thawed cartilage is generally accepted protocol in the imaging studies. Furthermore, since only ICRS-scoring was conducted and no quantitative measurements at e.g. light scattering were conducted, we believe that this had no effect on the conclusions of the present study.

In the present study, joints of cadaver horses were successfully evaluated by means of OCT. However, the sensitivity, reliability and reproducibility of arthroscopic OCT evaluation of joints *in vivo* need to be evaluated.

## Conclusions

OCT enhances intra- and inter-investigator agreement of arthroscopic evaluation of equine joints.

## Abbreviations

OCT: Optical coherence tomography; ICRS: International cartilage repair society.

## Competing interests

None of the authors of this paper has a financial or personal relationship with other people or organizations that could inappropriately influence or bias the content of the paper.

## Authors’ contributions

TN carried out arthroscopy, OCT imaging and writing of the manuscript. TV and JL participated in arthroscopies and OCT imaging and critically reviewed the manuscript. DA and NCRteM participated in scoring of the images and critically reviewed the manuscript. PHP participated in arthroscopies and OCT imaging and critically reviewed the manuscript. JSJ critically reviewed the manuscript. R-MT participated in the design of the study and helped to draft the manuscript. JT conceived of the study, and participated in its design and arthroscopies and OCT imaging, and helped to draft the manuscript. All authors read and approved the final version of the manuscript.
